# Electroosmotic Flow in Microchannel with Black Silicon Nanostructures

**DOI:** 10.3390/mi9050229

**Published:** 2018-05-11

**Authors:** An Eng Lim, Chun Yee Lim, Yee Cheong Lam, Rafael Taboryski

**Affiliations:** 1 School of Mechanical and Aerospace Engineering, Nanyang Technological University, 50 Nanyang Avenue, Singapore 639798, Singapore; lima0028@e.ntu.edu.sg; 2 Engineering Cluster, Singapore Institute of Technology, 10 Dover Drive, Singapore 138682, Singapore; chunyee.lim@singaporetech.edu.sg; 3 Department of Micro- and Nanotechnology, Technical University of Denmark, 2800 Kongens Lyngby, Denmark; rata@nanotech.dtu.dk

**Keywords:** micro-/nanofabrication, reactive ion etching, injection molding, electroosmotic flow, current monitoring method, finite element method

## Abstract

Although electroosmotic flow (EOF) has been applied to drive fluid flow in microfluidic chips, some of the phenomena associated with it can adversely affect the performance of certain applications such as electrophoresis and ion preconcentration. To minimize the undesirable effects, EOF can be suppressed by polymer coatings or introduction of nanostructures. In this work, we presented a novel technique that employs the Dry Etching, Electroplating and Molding (DEEMO) process along with reactive ion etching (RIE), to fabricate microchannel with black silicon nanostructures (prolate hemispheroid-like structures). The effect of black silicon nanostructures on EOF was examined experimentally by current monitoring method, and numerically by finite element simulations. The experimental results showed that the EOF velocity was reduced by 13 ± 7%, which is reasonably close to the simulation results that predict a reduction of approximately 8%. EOF reduction is caused by the distortion of local electric field at the nanostructured surface. Numerical simulations show that the EOF velocity decreases with increasing nanostructure height or decreasing diameter. This reveals the potential of tuning the etching process parameters to generate nanostructures for better EOF suppression. The outcome of this investigation enhances the fundamental understanding of EOF behavior, with implications on the precise EOF control in devices utilizing nanostructured surfaces for chemical and biological analyses.

## 1. Introduction

Electroosmotic flow (EOF) is a fluid flow in a micro-/nano-sized channel driven by an applied electric field as a result of an electrokinetic phenomenon. When a solid surface is in contact with an electrolyte solution, the spontaneous formation of surface charge attracts counterions and repels coions in the solution, which brings about a thin net charge layer (nanometer thickness) known as the electrical double layer (EDL). Upon application of an electric field parallel to the wall surface, the electrical body force acts on the excess counterions in the EDL that drives its motion, which is transferred to the bulk fluid through viscous effect. The fluid velocity is given by the Helmholtz-Smoluchowski equation (also called the slip velocity equation):

*v_eo_* = −*ε_r_ε_o_Eζ*/*µ* = *u_eo_E*, (1)

where *ε_r_* is the relative permittivity of fluid, *ε_o_* is the permittivity of free space, *E* is the applied electric field, *ζ* is the zeta potential, *µ* is the fluid viscosity and *u_eo_* is the EOF mobility.

Equation (1) is only valid if the size of the fluidic channel is large as compared to the thickness of EDL. The EDL thickness is represented by the Debye length (for a symmetric electrolyte):

*λ_D_* = (*ɛ_r_ɛ_o_k_b_T*/2*z*^2^*e*^2^*N_a_c_o_*)^1/2^, (2)

where *k_b_* is the Boltzmann constant, *T* is the temperature, *z* is the absolute charge number of the main constituent ion species, *e* is the electron charge, *N_a_* is the Avogadro constant and *c_o_* is the concentration of solution.

EOF has been used for numerous microfluidic applications such as electroosmotic pumping [1,2], electrokinetic mixing [3,4,5], chemical species/particles separation [6,7], preconcentration of biomolecules [8,9] and disease diagnosis from blood [10]. Microelectrodes are often utilized for EOF applications such as AC electroosmotic pumps to improve the flow rate and frequency range [11]. As opposed to the conventional techniques that require multiple fabrication steps and registration, i.e., alignment of the electrodes with the microfluidic channels, which are technically challenging, the fabrication of microelectrodes can be easily achieved by using dielectrophoresis for creating 3-D Galinstan microstructures [12], and injecting EGaIn liquid metal into microstructures that are aligned with and in direct contact with the fluidic channels [13].

Even though EOF has been extremely useful in the aforesaid applications, it can have very dramatic and undesirable consequences on the performance of certain applications. For instance, EOF degrades the resolution of electrophoresis analysis [14,15] because it produces a counterflow in the direction opposite to the electromigration of negatively charged biomolecules such as deoxyribonucleic acid (DNA) and sodium dodecyl sulfate (SDS) denatured proteins. Another example will be ion preconcentration, such as field-amplified sample stacking (FASS) [16] and isotachophoresis (ITP) [17], where the non-uniform EOF velocities due to the mismatch in solution conductivities generate internal pressure gradients that cause unwanted sample dispersion, which affects the sensitivity and resolution of such applications.

Conventionally, EOF is suppressed or eliminated by polymer coatings [18,19], e.g., acidified poly(ethylene oxide) (PEO) [20,21] and polyvinyl alcohol (PVA) [22,23]. However, polymer coatings can potentially contaminate the working solution and affect the analysis resolution of separation techniques [24]. Some polymer coatings involve complicated preparation procedure [23] and the stability of the coatings are compromised under extreme conditions, e.g., basic conditions and high organic solvent content in a running buffer [25].

Nanostructures are commonly introduced in microchannels for various purposes, e.g., electrophoretic separation of biosamples [26], high efficiency microreactors [27,28], facilitation of heat transfer [29,30] and enhancement of sensing capability [31,32]. The presence of nanostructures in microchannels has been known to reduce EOF, when the nanostructures are against the EOF flow direction [33,34,35,36,37]. The suppression of EOF by a nanopillar array was first reported by Yasui et al. [33], who found that the EOF mobility inside a nanopillar region with 700 nm spacing decrease by one order of magnitude when compared to the region outside. Our previous investigation [37] revealed that nanolines which are perpendicular to the EOF flow direction reduce EOF by approximately 20% due to the distortion of local electric field in the vicinity of the nanolines. Koga et al. [34] discovered that by increasing the surface roughness *R_a_* from approximately 30 nm to 300 nm, a decrease in EOF velocity from 900 µm/s to 500 µm/s could be obtained. Therefore, the idea of integrating nanostructures in a microchannel can serve as an alternative for EOF suppression.

However, the fabrication of large-area nanostructures with good regularity in a microchannel is technically challenging and very costly. For the study carried out by Yasui et al. [33], electron beam lithography (EBL) (sub-20 nm resolution) [38,39] was used to define the nanopillar array structures which were then transferred to the microchannel created by photolithography, for the investigation of its effect on EOF suppression. However, the conventional EBL is too costly and time consuming for large-area patterning, especially for single-use fluidic chips.

In our previous work [37], we adopted the deep ultraviolet (DUV) [40,41] lithography (sub-50 nm resolution) to construct parallel/perpendicular nanolines (within a microchannel) on a silicon mold. A nickel insert was then fabricated from the silicon mold via electroplating. With the insert, polymeric microfluidic channels with nanolines can be mass-produced by injection molding. This method allows the fabrication of large-area micro-/nanoscale patterns with good regularity in large quantities. However, photomasks of different dimensions for the nanostructures have to be fabricated for the DUV lithography step when their dimensions need to be varied, which is expensive. This makes the fabrication process rather inflexible. 

Koga et al. [34] altered the surface roughness (on a nanometer scale) of glass material by wet/dry etching processes, i.e., hydrofluoric acid (HF) etching and neutral loop discharge (NLD) plasma etching. Thereafter, a polydimethylsiloxane (PDMS) layer with the microchannel structure molded from photolithography patterned SU-8 was bonded to the etched glass plate through oxygen plasma treatment. This finding strongly suggests the possibility of employing etching process to gain controllability over the nanoscale patterns produced, through controlling the etching conditions.

Black silicon nanostructures [42,43], commonly known as silicon nanograss, have been widely used in many applications including solar panel and image sensor. The structures are produced by reactive ion etching (RIE) through the combined effect of an etching gas (SF_6_ and/or CH_4_) and a passivating gas (O_2_) [44,45,46]. Hitherto, no investigation has been carried out with RIE for the creation of large-area nanostructures within microchannel. This maskless method can solve the flexibility issue of the existing micro-/nanofabrication techniques with the ability to create large-area of moderately regular nanostructures.

In this investigation, we propose the employment of a fully scalable Dry Etching, Electroplating and Molding (DEEMO) process [47,48,49,50] for the fabrication of active microfluidic devices comprising nanostructures to control the EOF effect. This is critical for biological applications when the polymer coatings used to prevent nonspecific adhesion of cells are unable to suppress EOF, e.g., bovine serum albumin (BSA) coated microchannel exhibits significant zeta potential in phosphate buffered saline with sheep erythrocytes [51]. Most importantly, the nanostructures are situated at the channel wall in our device, which prevents the trapping of biomolecules such as DNA during biological analyses. The nanostructures were replicated from large-area black silicon nanostructures originated by RIE of silicon. The micro-/nanofluidic devices were replicated by injection molded with cyclic olefin copolymer (COC) and sealed by thermal bonding [48,52]. The effect of black silicon nanostructures on EOF in a microfluidic channel was then examined experimentally by current monitoring method, and numerically by finite element simulations. 

## 2. Materials and Methods

### 2.1. Fabrication Methods

#### 2.1.1. Microchannel Designs with/without Black Silicon Nanostructures 

**Figure 1:**
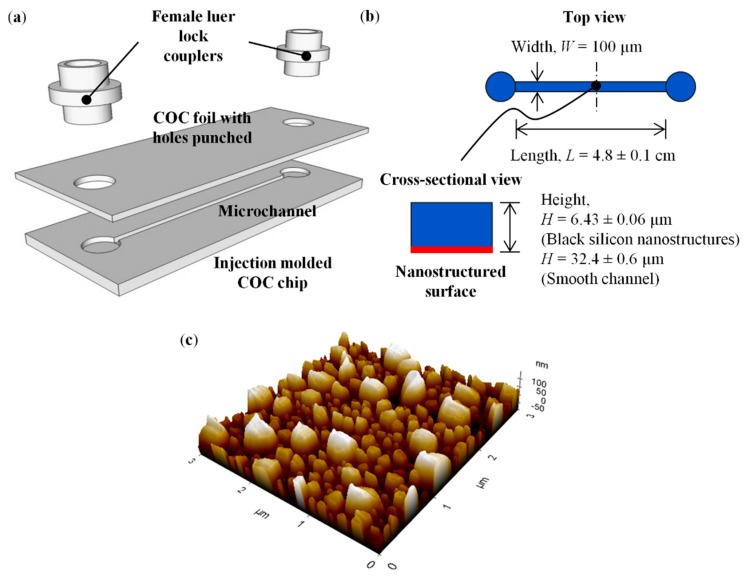
(**a**) 3-D exploded view diagram of micro-/nanofluidic device. (**b**) Schematic of microchannel designs with/without black silicon nanostructures. (**c**) Atomic force microscope (AFM) image of black silicon nanostructures on the bottom wall of the microchannel.

Three-dimensional (3-D) exploded view diagram of the micro-/nanofluidic device is shown in Figure 1a. The injection molded COC chip had dimensions of 75 mm × 25 mm × 1 mm, which resembles that of a standard microscope slide. The injection molded chip was thermal bonded with a COC foil of approximately 100 µm for enclosure of the open microchannel, and the thin COC foil fulfils the need of short working distance for high magnification microscopy experiments. To ensure easy fluidic access for the system, female luer lock couplers were employed to serve as practical inlet/outlet ports.

The microchannel design is shown in Figure 1b. The rectangular microchannel had width of 100 µm and length of 4.8 ± 0.1 cm, with large-area of black silicon nanostructures on the bottom wall of the channel. The black silicon nanostructures were prolate hemispheroid-like structures with diameter of 270 ± 73 nm, height of 175 ± 22 nm and spatial distance of 350 ± 89 nm. Characterization of the nanostructures was performed via an atomic force microscope (AFM, Park NX20) with NANOSENSORS™ PPP-NCHR probe (tip radius curvature <10 nm, tip length 10–15 µm and opening angle 15–20°). The AFM image of the black silicon nanostructures is as shown in Figure 1c. A smooth microchannel without any nanostructure (roughness arithmetic mean value *Ra* = 5.5 ± 0.6 nm) was fabricated to serve as a reference for zeta potential and EOF velocity measurements. The height of the microchannel with black silicon nanostructures was 6.43 ± 0.06 µm, and the height of the smooth microchannel was 32.4 ± 0.6 µm, as measured by a Dektak-XT stylus surface profiler. The additional channel height of the smooth channel ensured ease of fabrication without affecting the EOF flow velocity because EOF produces the same velocity regardless of the cross-sectional area [53,54]. 

#### 2.1.2. DEEMO Process for the Fabrication of Master Structures on Silicon Wafers

**Figure 2:**
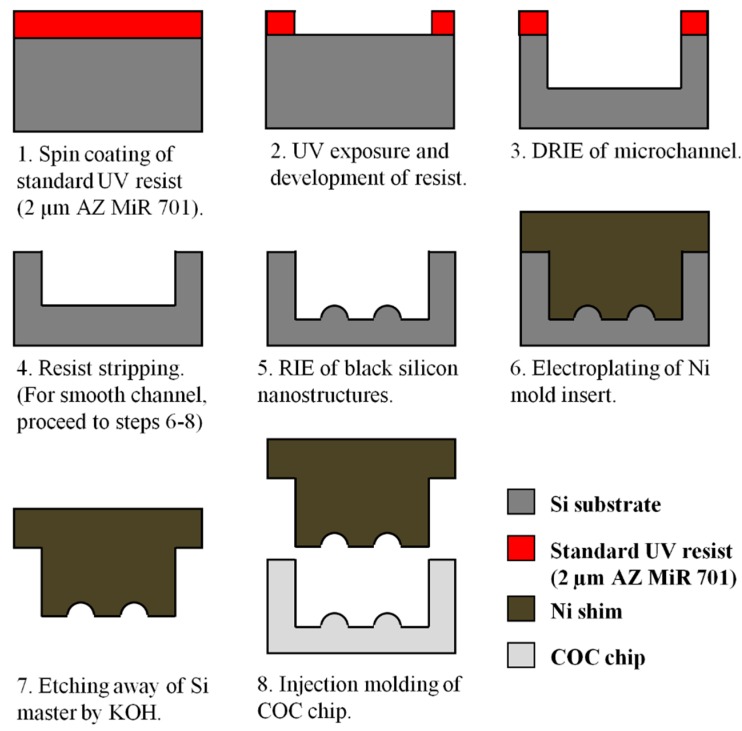
Schematics of Dry Etching, Electroplating and Molding (DEEMO) fabrication process for microchannel with large-area of black silicon nanostructures and smooth microchannel.

The fabrication of silicon master for microchannel with black silicon nanostructures requires a two-step etching process (see Figure 2). The silicon wafer was vacuum baked for 30 s and hexamethyldisilazane (HMDS) primed for 72 s to remove the native oxide, followed by spin coating of a 2 µm thick layer of positive resist (AZ MiR 701, MicroChemicals, Ulm, Germany), and then prebaking at 90 °C for 1 min via the SGV 88 track system. A chrome mask was used to define the flow channel pattern (width of 100 µm and length of 5 cm), and transferred onto the wafer through standard UV lithography via Karl Süss MA6/BA6 contact aligner (350 W Hg lamp with i-line filter as light source, exposure wavelength of 365 nm) in hard contact mode with exposure dose of 175 mJ/cm^2^. Next, the exposed wafer was post-baked at 110 °C for 1 min and then puddled in tetramethylammonium hydroxide (TMAH) developer for 1 min (Süss MicroTec Gamma system, Garching, Germany). 

Deep reactive ion etching (DRIE) with repetitions of alternating C_4_F_8_/SF_6_/O_2_ etching and C_4_F_8_ passivation cycles was used for the etching of microchannel to a desired depth (STS Pegasus system, Newport, UK). For microchannel with black silicon nanostructures, the wafer was exposed to C_4_F_8_ >> C_4_F_8_/SF_6_/O_2_ = 50 >> 10/70/5 sccm (passivation >> etch) with coil/platen power = 600/0 >> 400/40 W, pressure = 10 mtorr, cycle time = 2.5 >> 5 s, cycles = 50 and temperature = 20 °C. Subsequently, resist stripping was carried out in TePla 300 plasma asher for 10 min with flow rate of O_2_ at 400 sscm, N_2_ at 70 sscm, pressure of 1 mbar and power of 1000 W.

For the fabrication of the smooth microchannel, we employed the injection molding insert from our previous investigation [37]. The fabrication conditions were C_4_F_8_ >> SF_6_/O_2_ = 150 >> 275/15 sccm (passivation >> etch) with coil/platen power = 2000/0 >> 2500/35 W, pressure = 20 >> 26 mtorr, cycle time = 1 >> 2.2 s, cycles = 75 and temperature = 0 °C. Fabrication of silicon master for the smooth microchannel will be completed after this step, which is indicated as step 4 of Figure 2.

For the other (non-smooth) microchannel, an additional reactive ion etching (RIE) step was performed to produce large-area of black silicon nanostructures on the bottom wall of the etched channel [46]. The wafer was exposed to SF_6_/O_2_ plasma = 70/90 sccm with coil/platen power = 2600/25 W, pressure = 38 mtorr, time = 8 min and temperature = −10 °C, to generate the prolate hemispheroid-like nanostructures. Electroforming of the negative mold inserts for injection molding will be discussed in details in the subsequent section.

#### 2.1.3. Electroplating of Negative Mold Inserts

As silicon is brittle in nature, it is necessary to fabricate a metal mold insert, which can withstand several thousand cycles, for the polymer injection molding process. Therefore, negative mold inserts were fabricated by electroplating nickel (Ni) on the silicon masters produced in Section 2.1.2. A seed layer of approximately 85 nm nickel/vanadium (Ni/V, 93/7 wt %) was sputtered on the silicon wafers (Custom system, Kurt J. Lesker). This was to ensure that the surface being deposited on was conductive, in order for electroplating to be carried out. The slight amount of vanadium added could prevent the oxidation of nickel, which would otherwise affect the electroplating process. Subsequently, the sputtered wafers were immersed in nickel bath (Microform 200, Technotrans, Gersthofen, Germany) for approximately 6 h to electroplate shims of 350 μm thickness (maximum current of 3.5 A and charge of 18.1 Ah) [48,52]. 

Potassium hydroxide (KOH) etching was performed to remove the silicon masters from the Ni electroforms for approximately 8 h in 25 wt % KOH at temperature of 80 °C because of the strong adhesion. The resulting electroforms were cut into shims of 85 mm diameter by 50 W picosecond laser (FUEGO, Time-Bandwidth, Milpitas, CA, USA). Two flats were also cut on the Ni shims to define the orientation of the pattern when mounted in the injection molding tool [55]. The mold inserts were pre-coated with perfluorodecyltrichlorosilane (FDTS) monolayer (MVD100E, Applied Microstructures, San Jose, CA, USA), which served as an anti-stiction layer [56].

#### 2.1.4. Polymer Injection Molding

An Engel Victory 80/45 Tech hydraulic injection molder equipped with an Engel ERC 13/1-F pick up robot was used. TOPAS 5013L-10 COC (Topas Advanced Polymers GmbH, Frankfurt-Höchst, Germany) which has a high flowability, an important attribute for the filling of nanoscale structures on the mold inserts, was employed for the polymer injection molding process [55]. The injection molded COC microchannels with/without black silicon nanostructures have excellent biocompatibility for biological applications such as biosensing and bioparticle sorting which involve interaction with proteins, cells and blood components etc., because it has been shown that different grades of COC exhibit no signs of in vitro cytotoxicity [57]. In addition, it has a relatively high glass transition temperature of 135 °C which is suitable for biological experiments at elevated temperatures, e.g., DNA denaturation [58] and DNA amplification by polymerase chain reaction (PCR) [59]. 

A variotherm process was adopted for the injection molding of microchannel with black silicon nanostructures, while a constant temperature process was used for the molding of smooth microchannel instead [60]. Detailed parameters for the polymer injection molding of microchannels with/without black silicon nanostructures are shown in Table S1 of the Supplementary Materials.

#### 2.1.5. Thermal Bonding and Integration of Practical Inlet/Outlet Ports

The injection molded COC chips were thermal bonded to an extruded 101.6 μm thick TOPAS 5013L-10 COC foil by Specac Atlas manual hydraulic press with heated platens. Entrance and exit holes of approximately 4.8 cm apart were punched on the COC foil, and aligned with the open microchannel on the molded chip (see Figure 1a). The chip and foil were sandwiched between two PDMS layers (approximately 3 mm thick each) to compensate for the slight non-uniformities in the platen flatness, so that uniform pressure could be exerted across the chip surface. A piston force of 0.25 kN was applied for 15 min with temperature of 128 °C to bond the chip to the foil.

To connect the microchannel to external flow or air pressure control, practical inlet/outlet ports were incorporated to the system through the employment of white nylon female luer lock couplers (Cole-Parmer) (see Figure 1a). The luer lock couplers were first attached to the entrance and exit holes by Pro-Spec epoxy steel adhesive with mixing ratio 1:1 and set time of 4 min. Then, UV adhesive (Dymax 215-CTH-LV-UR-SC) was used to adhere the couplers to the chip. The purpose of applying the epoxy steel adhesive first was to prevent the low viscosity UV adhesive from clogging the inlet and outlet of the microchannel. An UV flood curing system (UVF600, Technodigm, Singapore) was utilized for the curing of UV adhesive with a curing time of 8 min. Thereafter, the devices were left under normal room conditions for 24 h, so that the adhesive could reach its full strength.

### 2.2. Current Monitoring Experiments

**Figure 3:**
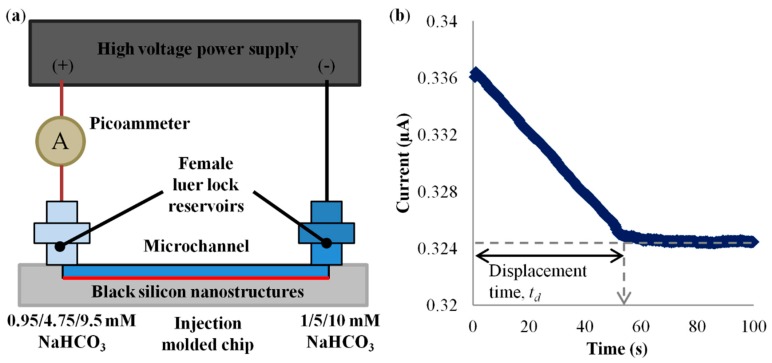
(**a**) Experimental setup for current monitoring technique. (**b**) Current-time curve for 0.95 mM NaHCO_3_ displaced 1 mM NaHCO_3_ in smooth microchannel.

Current monitoring technique [61,62,63,64] was performed to investigate the effect of black silicon nanostructures in microchannel on EOF. Figure 3a shows the schematic diagram of the experimental setup. The electric field for inducing EOF was supplied by a high voltage power supply (CZE1000R, Spellman, Hauppauge, NY, USA) through platinum electrodes. The current across the microchannel was monitored by connecting a picoammeter (Keithley 6458) in series to the microchannel. A Labview program was written to control the two devices, and to record the voltage and current readings through a data acquisition card (PCI-6014, National Instrument, Austin, TX, USA).

The effect of black silicon nanostructures on EOF was characterized by conducting experiments with sodium bicarbonate solutions (NaHCO_3_) of different concentrations, i.e., 1 mM, 5 mM and 10 mM, in comparison to the smooth microchannel. The solutions were prepared by dissolving the NaHCO_3_ salt (Sigma-Aldrich, Saint Louis, MO, USA) in ultrapure (Type 1) water (Direct-Q 5 UV, Merck, Kenilworth, NJ, USA). Table 1 presents the measured conductivities (IONCheck 65, Radiometer Analytical, Loveland, CO, USA) and pH values (FEP20, Mettler Toledo, Singapore) for the solutions used in the experiments.

Electric potential of 500 V was applied to generate EOF. Through the application of EOF, the microchannel was flushed with 1/5/10 mM NaHCO_3_ for at least 30 min to make certain the complete filling of the nanostructures to ensure consistency and repeatability. Thereafter, the microchannel was filled with fresh 1/5/10 mM NaHCO_3_ for the current monitoring experiment. The microchannel and cathode reservoir were filled with 1/5/10 mM NaHCO_3_, while the anode reservoir was filled with 0.95/4.75/9.5 mM NaHCO_3_ (95% concentration of the solution to be displaced) (see Figure 3a). The time for the current to reach a steady current value, i.e., displacement time, was deduced from the current-time curve (see Figure 3b). The average EOF velocity was then calculated by dividing the length of the microchannel with the displacement time:

*v_avg_* = *L*/*t_d_*, (3)

where *L* is the length of microchannel and *t_d_* is the displacement time.

EOF of two solutions with dissimilar ionic species [65,66] or large concentration difference [67,68,69] causes accumulation/depletion of the main constituent ionic species or pH-governing minority ions, which induces pH changes and demonstrates hysteresis phenomenon, whereby the EOF flow rate for solution A displacing solution B is different from that of solution B displacing solution A. Moreover, electrolysis at the electrodes generates hydronium (H_3_O^+^) and hydroxide (OH^−^) ions that will alter the pH in the reservoirs [70], which in turn affect the EOF velocity. Hence, several precautions were implemented to ensure negligible pH change during the experiments.

NaHCO_3_ buffered solutions were employed to ensure the pH was constant throughout the experiments. Small concentration difference (5% difference) between the displacing and residing solutions was used to minimize the pH changes during the displacement process. The large-volume reservoirs (200 µL) diluted the concentrations of H_3_O^+^ and OH^−^ ions produced at the electrodes from electrolysis significantly, which at the same time ensured negligible liquid level changes to minimize the back pressure generated [71]. The short experimental durations (less than 100 s) and small electrical currents (0.0536–3.16 µA) due to the small microchannel cross-sections (640/3200 µm^2^) and low solution conductivities (96.2–912.8 µS/cm), also restricted the production of H_3_O^+^ and OH^−^ ions [72]. pH indicator strips (Merck 109535) were used for the pH measurement in the reservoirs, both before and after the experiments, and the pH changes were found to be insignificant.

The effect of Joule heating that distorts the usual plug-like velocity profile of EOF can be neglected because of the low conductivities of the solutions (96.2–912.8 µS/cm) [73]. A conservative estimate of Joule heating can be calculated from the energy balance between energy generation *Eg* and energy storage ∆*Est* in the liquid [74]. For the chosen experimental parameters, the worst case scenario (applied electric field = 104 V/cm, NaHCO_3_ concentration = 10 mM, microchannel cross-sectional area = 640 µm^2^ and experimental duration = 100 s) has an estimated temperature rise of 1 °C, which is negligible.

### 2.3. Numerical Simulations

**Figure 4:**
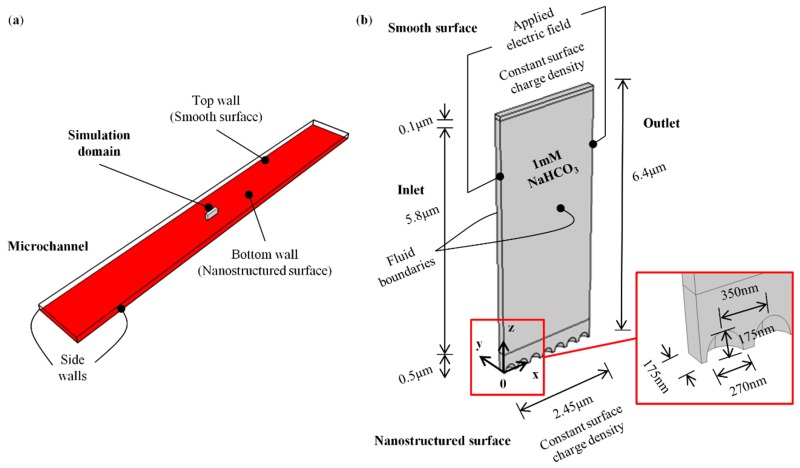
(**a**) Fluid segment sliced from microchannel for simulation. (**b**) 3-D simulation domain.

Numerical simulations based on finite element method (FEM) were performed on steady-state EOF of 1 mM NaHCO_3_. Poisson-Nernst-Planck (PNP) model with modified boundary conditions [37,65,66,68] was employed. 3-D simulation was implemented on COMSOL Multiphysics for the investigation of the effect of black silicon nanostructures on EOF in the microchannel. As the actual nanostructures are not entirely uniform, we had measured the average parameters, such as diameter, height and density through AFM. In this numerical study, we approximated the nanostructures as a regular series of prolate hemispheroids with the experimentally measured average parameters: diameter *d* = 270 nm, height *h* = 175 nm and spatial distance *s* = 350 nm. Simulating the entire microchannel with 3-D nanostructures requires extreme computational resources. Therefore, a short segment of fluid phase, sliced from top to bottom of the microchannel was simulated instead (see Figure 4a). The top and bottom walls of the microchannel dictate the flow velocity while the side walls have negligible effect on fluid flow because the actual microchannel width (100 μm) is much larger than its height (6.43 μm). 

A voltage of 0.0255 V was set at the inlet of the simulation domain (see Figure 4b) to establish an electric field of 104 V/cm, which is similar to the experiment (see Section 2.2). The surface charge density *S* of the smooth and nanostructured surfaces was specified as −1.54 × 10^−2^ C/m^2^ (calculated by Grahame equation [54]) based on the nominal “material” zeta potential (−108.7 mV, effective zeta potential of 1 mM NaHCO_3_ for smooth channel) of the COC fluidic devices (see Section 3). Boundary conditions for no mass and charge transfer across the physical boundaries were set at the smooth and nanostructured surfaces. Since only half of the prolate hemispheroids were simulated (see Figure 4b) to reduce the computational effort, the pattern does not demonstrate a periodically repeating nature that qualifies for the use of periodic boundary conditions. Instead, symmetric boundary conditions were prescribed at the fluid boundaries (side surfaces of the simulation domain) for which the electric field, fluid velocity, and ion fluxes travel parallel to the boundaries. Detailed boundary conditions for the 3-D numerical simulation of a steady-state EOF is shown in Table S2 of the Supplementary Materials.

The simulation domain was designed to consist of three parts (see Figure 4b), namely *y* = 0 µm to *y* = 0.5 µm, *y* = 0.5 µm to *y* = 6.3 µm and *y* = 6.3 µm to *y* = 6.4 µm, for the control of the mesh type and size. The domain was meshed with 733,969 tetrahedral elements from *y* = 0 µm to *y* = 0.5 µm, and 2496 triangular prism elements from *y* = 0.5 µm to *y* = 6.3 µm and *y* = 6.3 µm to *y* = 6.4 µm. The size of the elements at the nanostructured wall surface was set to a maximum of 5 nm. The size of the mesh increases gradually with increasing distance from the wall (at a maximum growth factor of 2), while the element size at the smooth surface was prescribed to be less than 2 nm. The rationale for the higher mesh density near the wall surface is to resolve the steep changes of variables across the EDL. 2-D simulation was conducted for the smooth microchannel to serve as a comparison to the microchannel with black silicon nanostructures. Details on the 2-D simulation domain, boundary conditions and mesh selection can be found in our previous work [37].

The governing equations for the PNP model are:

▽·(*σ*▽*φ*) = 0, (4)

▽·▽*ψ* = −*ρ_e_*/*ɛ_r_ɛ_o_*, (5)

*∂c_i_*/*∂t* + ▽·[−*D_i_*▽*c_i_* − *u_m(i)_c_i_*▽(*φ* + *ψ*)] = −***v***·▽*c_i_*, (6)

*ρ∂**v***/*∂t* = −▽*p* + *µ*▽^2^***v*** + *ρ_e_*[−▽(*φ*)], (7)

▽·***v*** = 0. (8)

where *σ* = *F*∑*z_i_u_m(i)_c_i_* is the solution conductivity, *φ* is the applied electric field, *F* is the Faraday constant, *D_i_* is the diffusion coefficient, *u_m(i)_* is the ionic mobility, *z_i_* is the charge number, *c_i_* is the concentration of the ionic species, *ρ_e_* = *F*∑*c_i_z_i_* is the net charge density, *ψ* is the electrostatic wall potential, *ρ* is the fluid density, *p* is the pressure and ***v*** is the fluid velocity.

In the numerical simulations for the generation of EOF, the Laplace equation (Equation (4)), Poisson equation (Equation (5)) and Nernst-Planck equation (Equation (6)) were solved simultaneously with the Navier-Stokes and continuity equations (Equations (7) and (8)). The applied potential, electrostatic wall potential, and ion concentrations were discretized with second order elements, while the pressure and velocity were discretized with linear elements. The convergence criterion was based on relative tolerance of less than 0.001 between subsequent iterations. The symbols and values of constants employed for the numerical simulations can be found in Table S3 of the Supplementary Materials.

Numerical simulations were performed by systematically varying the height and diameter of the hemispheroid structures, to investigate the geometrical effect of the black silicon nanostructures on EOF in a microchannel. The variations of structure height *h* were 87.5 nm (50% reduction in the experimental height), 175 nm (employed in the current investigation) and 262.5 nm (50% increment in the experimental height), while the structure diameter *d* and spatial distance *s* were fixed at 270 nm and 350 nm respectively. The variations of structure diameter *d* were 135 nm (50% reduction in the experimental diameter), 270 nm (employed in the current investigation) and 337.5 nm (25% increment in the experimental diameter), while the structure height *h* and spatial distance *s* were fixed at 175 nm and 350 nm respectively.

## 3. Results and Discussion

**Figure 5:**
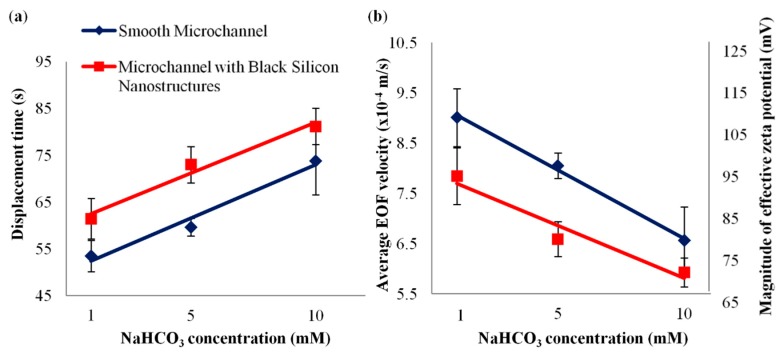
(**a**) Displacement times, (**b**) electroosmotic flow (EOF) velocities and magnitude of effective zeta potentials for 1 mM, 5 mM and 10 mM of NaHCO_3_ for microchannel with black silicon nanostructures, in comparison to smooth microchannel.

The displacement times for 1 mM, 5 mM and 10 mM of NaHCO_3_ in microchannel with black silicon nanostructures were measured with current monitoring technique (see Section 2.2), in comparison with the smooth microchannel (see Figure 5a). The measured displacement times for 1 mM, 5 mM and 10 mM of NaHCO_3_ for microchannel with black silicon nanostructures were 61 ± 4 s, 73 ± 4 s and 81 ± 4 s respectively, and for the smooth microchannel were 54 ± 3 s, 60 ± 2 s and 74 ± 7 s respectively. 

In both cases, the displacement times increased with increasing NaHCO_3_ concentration (see Figure 5a). The Debye length decreased from approximately 10 nm to 4 nm and to 3 nm, when the NaHCO_3_ concentration was increased from 1 mM to 5 mM and to 10 mM respectively (as calculated by Equation (2)). The decrease in Debye length was because there was higher concentration of ion species for the screening of surface charge, which resulted in a lower zeta potential that reduced the EOF velocity (see Equation (1)) and caused a longer displacement time. However, as compared to the smooth microchannel, the displacement times for microchannel with black silicon nanostructures were increased by approximately 16 ± 9% for the three NaHCO_3_ concentrations (see Figure 5a). 

The average EOF velocities were calculated through Equation (3) (see Figure 5b). The average EOF velocities for 1 mM, 5 mM and 10 mM of NaHCO_3_ for microchannel with black silicon nanostructures were (7.8 ± 0.6) × 10^−4^ m/s, (6.6 ± 0.3) × 10^−4^ m/s and (5.9 ± 0.3) × 10^−4^ m/s respectively, while the velocities for smooth microchannel were (9.0 ± 0.6) × 10^−4^ m/s, (8.1 ± 0.3) × 10^−4^ m/s and (6.6 ± 0.7) × 10^−4^ m/s respectively. It can be observed from Figure 5b that EOF velocity was reduced by approximately 13 ± 7% for microchannel with black silicon nanostructures. This concurs with the existing literature [33,34,35,36,37], which shows that the presence of nanostructures in microchannel (against the EOF flow direction) lowers the EOF velocity.

The concept of effective zeta potential [34,37] was adopted to incorporate both the effects of nanostructures and the nominal “material” zeta potential, for the revelation of the relationship between the EOF velocity and the surface topography. The EDL thickness (reflected by the Debye length) for different NaHCO_3_ concentrations were thin as compared to the micro-/nanostructures of the fabricated fluidic devices (see Section 2.1.1). Thus, the effective zeta potential which includes the surface topography and the chemical properties can be derived by substituting the calculated average EOF velocity into Equation (1), which can be expressed as:

*ζ_eff_* = −*v_avg_µ*/*ɛ_r_ɛ_o_E*. (9)

The effective zeta potentials (their magnitude) for 1 mM, 5 mM and 10 mM of NaHCO_3_ in microchannel with black silicon nanostructures, and the smooth microchannel are shown in Figure 5b. The effective zeta potentials for 1 mM, 5 mM and 10 mM of NaHCO_3_ for microchannel with black silicon nanostructures were 95 ± 7 mV, 80 ± 4 mV and 72 ± 4 mV respectively, and for smooth microchannel were 109 ± 7 mV, 97 ± 3 mV and 79 ± 8 mV respectively. It can be observed (see Figure 5b) that the effective zeta potential, and hence the EOF velocity, were lowered significantly by approximately 13 ± 7% with the introduction of black silicon nanostructures in the microchannel. 

**Figure 6:**
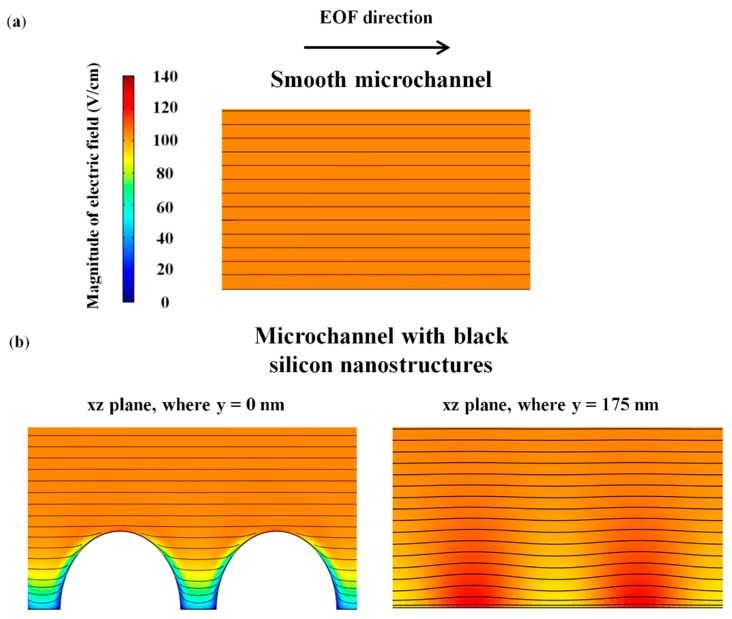
Simulated electric field lines for (**a**) smooth microchannel and (**b**) microchannel with black silicon nanostructures, for 1 mM of NaHCO_3_.

The underlying mechanics for EOF reduction by the black silicon nanostructures (prolate hemispheroid-like structures) was studied numerically through the finite element simulation (see Section 2.3). Similar as our previous investigation [37], EOF is reduced due to the distortion of electric field at the nanostructured wall surface which affects the effective zeta potential. Figure 6 shows that the presence of black silicon nanostructures distorts the local electric field near the vicinity of the nanostructured surface (see Figure 6b), which reduces the average electric field on the structured surface from 104 V/cm to 89.7 V/cm. 

**Figure 7:**
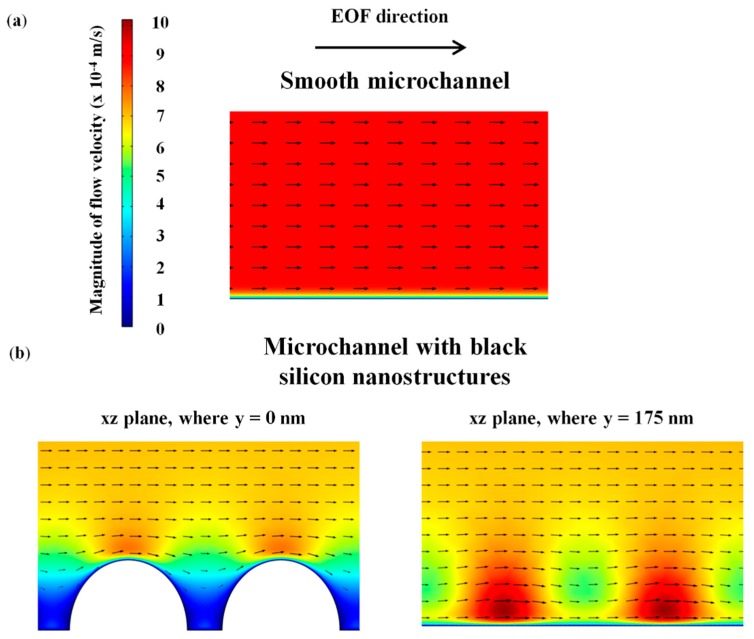
Simulated EOF velocity profile for (**a**) smooth microchannel and (**b**) microchannel with black silicon nanostructures, for 1 mM of NaHCO_3_.

**Figure 8:**
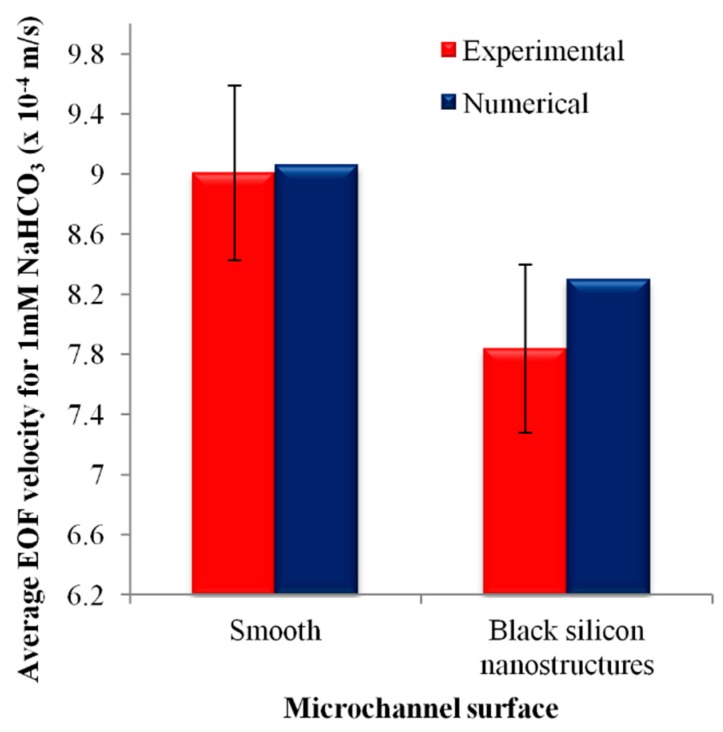
Experimental and numerical average EOF velocities of 1 mM NaHCO_3_ for microchannel with black silicon nanostructures, in comparison to smooth microchannel.

Since EOF is driven by the electric field at the wall surface, the reduction in the average electric field on the nanostructured surface reduces the fluid flow velocity near its vicinity (see Figure 7b), which affects the overall EOF velocity. The simulated average EOF velocity for 1 mM NaHCO_3_ decreases from 9.06 × 10^−4^ m/s to 8.30 × 10^−4^ m/s with the introduction of black silicon nanostructures (see Figure 8), which is approximately 8%. The simulation results underestimate the experimental results that showed an EOF flow velocity reduction of 13 ± 7% (see Figure 8). This could be due to the employment of a regular geometry for the black silicon nanostructures (diameter *d* = 270 nm, height *h* = 175 nm and spatial distance *s* = 350 nm) in the numerical simulation to approximate the physical nanostructures (*d* = 270 ± 73 nm, *h* = 175 ± 22 nm and *s* = 350 ± 89 nm) which are not entirely uniform. Despite this approximation, we showed that the simulated outcome is within the scatter of the experimental results (see Figure 8). The observed reduction of EOF in this preliminary investigation demonstrates the possibility of employing nanostructures produced through black silicon to suppress EOF.

**Figure 9:**
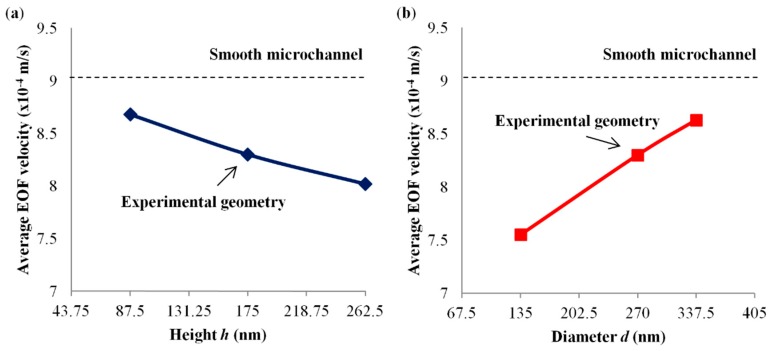
Variation of numerical average EOF velocity for 1 mM NaHCO_3_ with (**a**) height *h* where diameter *d* = 270 nm and spatial distance *s* = 350 nm, and (**b**) *d* where *h* = 175 nm and *s* = 350 nm.

The RIE process parameters, such as the flow rates of the etching gas (SF_6_ and/or CH_4_) and passivating gas (O_2_), can be varied to produce black silicon nanostructures of different dimensions [45,75]. The effect of geometry variations, i.e., height and diameter of the hemispheroid structures, on EOF was investigated by numerical simulations (refer to Section 2.3). Figure 9a shows that the simulated average EOF velocity decreases with increasing height of the black silicon nanostructures. The EOF velocity reduces by approximately 12% with 50% increment in the experimental structure height (see Figure 9a). Figure 9b shows a decrease of the stimulated average EOF velocity with decreasing diameter of the black silicon nanostructures. The decrease in EOF velocity is approximately 17% with 50% reduction in the experimental structure diameter (see Figure 9b). These simulated results on geometry variations reveal the potential of tuning the etching process parameters to generate nanostructures for control suppression of EOF.

## 4. Conclusions

In this investigation, we employed the DEEMO process along with reactive ion etching (RIE), for microchannel with black silicon nanostructures (prolate hemispheroid-like structures). The fabrication process consists of the following steps: (i) fabrication of master structures on silicon wafers, (ii) creation of mold inserts by electroplating, (iii) injection molding with COC, and (iv) thermal bonding and integration of practical inlet/outlet ports.

The effect of black silicon nanostructures on EOF in a microfluidic channel was examined experimentally by current monitoring method, and numerically by finite element simulations. The experimental results showed a reduction of 13 ± 7% in the effective zeta potential, and thus the EOF velocity, with the introduction of black silicon nanostructures in the microchannel. The simulation outcome which predicts an average EOF velocity reduction of approximately 8% is within the scatter of the experimental results. The EOF reduction is caused by the distortion of local electric field at the nanostructured surface, which decreases the average electric field, and hence reduces the overall EOF flow velocity.

The RIE process parameters can be varied to produce black silicon nanostructures of different dimensions. The effect of geometry variations on EOF was studied numerically. The average EOF velocity decreases with increasing structure height or decreasing structure diameter. The investigation on geometry variations demonstrates the potential of tuning the etching process parameters to generate nanostructures for control suppression of EOF in future investigations.

## Figures and Tables

**Table 1:** Measured conductivities and pH values of solutions used in experiments.

Solution (NaHCO_3_)	Conductivity (µS/cm)	pH
0.95 mM	96.2 ± 0.2	8.40 ± 0.01
1 mM	99.7 ± 0.1	8.32 ± 0.03
4.75 mM	436.6 ± 0.5	8.72 ± 0.02
5 mM	469.4 ± 0.5	8.71 ± 0.01
9.5 mM	851.2 ± 1.1	9.11 ± 0.01
10 mM	912.8 ± 0.4	9.14 ± 0.01

## Supplementary Materials

The following are available online at http://www.mdpi.com/2072-666X/9/5/229/s1, Table S1: Parameters for polymer injection molding of microchannels with/without black silicon nanostructures, Table S2: Boundary conditions for 3-D numerical simulation of a steady-state EOF, Table S3: Symbols and values of constants employed for numerical simulations where ionic mobility of an ion species is calculated by formula (*z_i_D_i_F*)/(*RT*).
