# Supplementary material for: Electroosmotic Flow in Microchannel with Black Silicon Nanostructures
Source: Micromachines (Basel). 2018 May 11;9(5):229. doi: 10.3390/mi9050229 (PMC6187698; doi:10.3390/mi9050229)
Supplement: Supplementary file 1 [file micromachines-09-00229-s001.pdf]

Supplementary Materials: Electroosmotic Flow in Microchannel with Black Silicon Nanostructures

Table S1. Parameters for polymer injection molding of microchannels with/without black silicon nanostructures.

Parameters	Constant Temperature Mode (Smooth Microchannel)	Variotherm Mode with Insert Cooling (Microchannel with Black Silicon Nanostructures)
Mold temperature	90 °C	130 °C
Melt temperature	270 °C	270 °C
Injection pressure	950 ± 25 bar	950 ± 25 bar
Injection velocity	37 mm/s	40 mm/s
Holding pressure	600 ± 25 bar for 3 s	400 ± 25 bar for 10 s
Cooling time/demolding temperature	30 s	95 ± 5 °C
Pre-drying (Permanent)	90 °C	90 °C
Cycle time	30 s to 1 min	2 min to 4 min

Table S2. Boundary conditions for 3-D numerical simulation of a steady-state EOF.

Variable	Condition	Boundary
Applied potential ϕ	$\phi = 0.0255$ V	Inlet
	$\phi = 0$ V	Outlet
	$-\mathbf{n}\cdot\sigma\nabla\phi = 0$ [a]	Smooth and nanostructured surfaces, and fluid boundaries
Electrostatic potential ψ	$\mathbf{n}\cdot\nabla\psi = 0$	Inlet, outlet and fluid boundaries
	$\mathbf{n}\cdot\nabla\psi = S/\varepsilon_r\varepsilon_o$	Smooth and nanostructured surfaces
Concentrations of ions c_i	$c_i = c_{o(i)}\exp(-z_i e\psi/k_b T)$	Inlet and outlet
	$-\mathbf{n}\cdot[-D_i\nabla c_i - u_{m(i)}c_i\nabla(\phi+\psi) + \boldsymbol{v}c_i] = 0$	Smooth and nanostructured surfaces, and fluid boundaries
Flow velocity $\boldsymbol{v}$ and Pressure p	$\boldsymbol{v} = 0$	Smooth and nanostructured surfaces
	$\mathbf{n}\cdot\boldsymbol{v} = 0$	Fluid boundaries
	$p = 0$	Inlet and outlet

[a] $\mathbf{n}$ is the unit vector normal to the boundary.

Table S3. Symbols and values of constants employed for numerical simulations where ionic mobility of a ion species is calculated by formula $(z_iD_iF)/(RT)$.

Parameters	Symbol (Unit)	Value
Permittivity of free space	ε_o (C/Vm)	8.85×10^{-12}
Relative permittivity	ε_r	80
Viscosity of water	μ (kg/ms)	8.90×10^{-4}
Density of water	ρ (kg/m^3)	1000
Faraday constant	F (C/mol)	96485
Gas constant	R (J/molK)	8.314
Boltzmann constant	k_b (m^{2}kg/s^{2}K)	1.381×10^{-23}
Temperature	T (K)	298
Electron charge	e (C)	1.602×10^{-19}
Avogadro constant	N_a (1/mol)	6.022×10^{23}
Solution concentration	c_o (mol/m^3)	1
Surface charge density	S (C/m^2)	-1.54×10^{-2}
Diffusion coefficient of Na^+	D_{Na^+} (m^2/s)	1.334×10^{-9}
Diffusion coefficient of HCO_3^-	D_{HCO3^-} (m^2/s)	1.105×10^{-9}
Ionic mobility of Na^+	$u_{m(Na^+)}$ (m^2/Vs)	5.194×10^{-8}
Ionic mobility of HCO_3^-	$u_{m(HCO3^-)}$ (m^2/Vs)	-7.919×10^{-8}
Ionic charge number of Na^+	z_{Na^+}	+1
Ionic charge number of HCO_3^-	z_{HCO3^-}	−1
